# Deep phylogeographic divergence of a migratory passerine in Sino-Himalayan and Siberian forests: the Red-flanked Bluetail (*Tarsiger cyanurus*) complex

**DOI:** 10.1002/ece3.967

**Published:** 2014-02-27

**Authors:** Site Luo, Yuchun Wu, Qing Chang, Yang Liu, Xiaojun Yang, Zhengwang Zhang, Min Zhang, Qiang Zhang, Fasheng Zou

**Affiliations:** 1 Guangdong Entomological Institute, South China Institute of Endangered AnimalsGuangzhou, 510260, China; 2 Institute of Genetic Resources, College of Life Sciences, Nanjing Normal UniversityNanjing, 210046, China; 3 State Key Laboratory of Biocontrol and School of Life Sciences, SunYat-sen UniversityGuangzhou, 510275, China; 4 State Key Laboratory of Genetic Resources and Evolution, Kunming Institute of Zoology, Chinese Academy of SciencesKunming, 650223, China; 5 Ministry of Education Key Laboratory for Biodiversity Sciences and Ecological Engineering, College of Life Sciences, Beijing Normal UniversityBeijing, 100875, China

**Keywords:** Sex chromosome, species delimitation, *Tarsiger cyanurus* complex, the phylogenetic species concept

## Abstract

Enormous mountainous forests in Sino-Himalayans and Siberia harbor important avian biodiversity in the Northern Hemisphere. Numerous studies in last two decades have been contributed to systematics and taxonomy of passerines birds in these regions and have revealed various and complex phylogeographic patterns. A passerine species Red-flanked Bluetail *Tarsiger cyanurus* provided a good system to manifest such evolutionary complexity. The subspecies *T. c. cyanurus* and *T. c. rufilatus* (or/and *T. c. pallidior*), divergent in morphology, acoustics, and migratory strategies are allopatric in Siberia and Sino-Himalayan forests, respectively. The two taxa most likely deserve full species status but rigorous genetic analysis is missing. In this study, multilocus phylogeography based on mitochondrial DNA and Z-linked DNA reveals that *T. c. cyanurus* and *T. c. rufilatus* are reciprocally monophyletic with significant statistical support and differ with a large number of diagnostic nucleotide sites resulting substantial genetic divergence. Our finding supports the proposed split of *Tarsiger cyanurus s.l*. that *T*.* cyanurus* and *T*.* rufilatus* should be treated as two full species. Whether “*pallidior”* is a subspecies or geographical form of *T*.* rufilatus* is still uncertain. Additionally, these two forest passerine species may have diverged 1.88 (3.25–1.30) Mya, which might be shaped by geographical vicariance due to grassland and desert steppe on the central Loess Plateau during the Pliocene. Taken together, this study and further suggests another independent example of North Palearctic–Sino-Himalayan phylogeographic pattern in Palearctic birds.

## Introduction

The uplifts of Tibetan Plateau, especially the most recent and abrupt uplift in 3.4 million years ago (Mya), have substantially influenced the phylogeographic patterns of the component fauna of the plateau as well as the adjacent areas including the Sino-Himalayan, the Siberia, and tropical Asia (Päckert et al. 2009, 2012; Yang et al. 2009; Price 2010; Martens et al. 2011). These large Eurasian mountain ranges in this area are considered an important hotspot of avian biodiversity in the Northern Hemisphere (Roselaar et al. 2007). Extensive ornithological studies on the systematics and taxonomy have been focused on many taxa distributed in this region, especially passerines (Päckert et al. 2009, 2012; Price 2010; Martens et al. 2011). Most of species distributed on/around the Tibetan Plateau and in North Palearctic regions demonstrated various degrees of differentiation in morphology, acoustics, ecology (i.e., migratory strategies), and genetics, which can been assigned to different geographical affinities, long been known as “subspecies”. Most of these divergences can be dated to the Pliocene–Pleistocene associated with geographical vicariance (Johansson et al. 2007; Päckert et al. 2009, 2012; Martens et al. 2011). Considering their long and independent evolution in geographical separation, however, some of them may merit treatment as “species” (Martens et al. 2011). Recent findings mainly based on the evidence of genetic or/and acoustic characters have revealed disconcordance between conventional taxonomy and phylogenetic relationships in many taxa (Cibois et al. 1999; Luo et al. 2009; Sangster et al. 2010; Zuccon and Ericson 2010; Martens et al. 2011). This has led to many taxonomic changes, especially the increase in species by splitting of long-established polytypic species or species complexes (Luo et al. 2009; Sangster et al. 2010; Zuccon and Ericson 2010; Martens et al. 2011), leading to the inflation the species-level diversity in Old World passerine birds.

One example of a passerine species complex distributed in the North Palearctic region and Sino-Himalayas is the Red-flanked Bluetail *Tarsiger cyanurus* Pallas (1773), which is placed in Old World flycatcher family Muscicapidae (Zhao 2001; Dickinson 2003; del Hoyo et al. 2005; Sangster et al. 2010). This species is a small migratory insectivorous bird that occupies a large breeding range in Eurasia, from northern Finland to Kamchatka and Japan in the Northern Palearctic, the northern, western, and southern Himalayas, and southwestern China. The Red-flanked Bluetail occurs in Southern China, Myanmar, Indochina, and the Himalayan foothills during nonbreeding period. It is sexually dimorphic and shows a habitat preference for both coniferous and broad-leaf mixed forests, subalpine forests and up to treeline in the Himalayas (del Hoyo et al. 2005).

**Figure 1:**
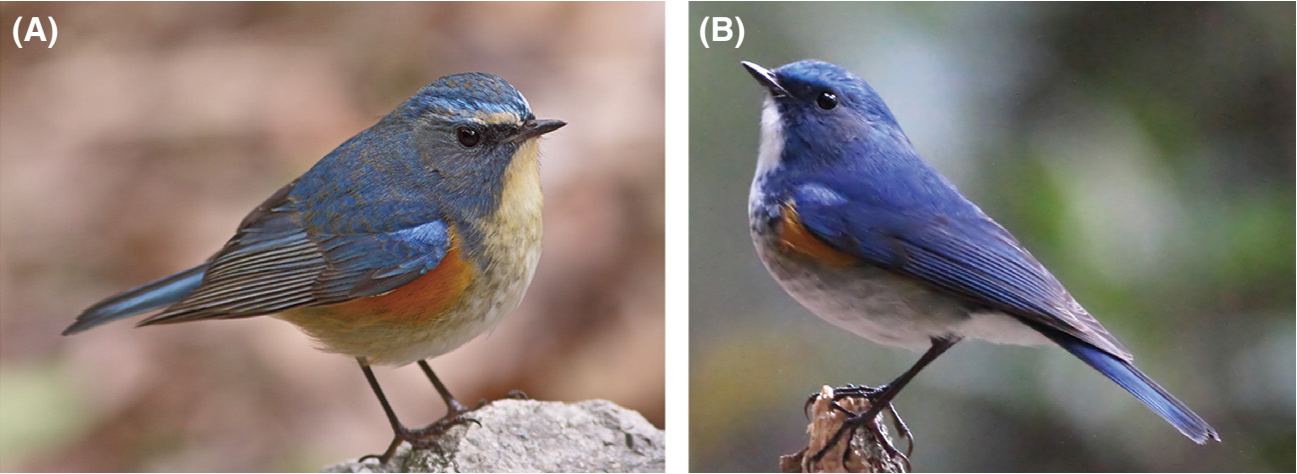
(A) Male *Tarsiger cyanurus cyanurus*. (B) Male *T. c. rufilatus*. (B) can be morphologically distinguished from (A) by darker blue tone and light blue supercilium (photographs by Menxiu Tong/China Wild Tour).

The Red-flanked Bluetail is currently split into two or three subspecies based on morphological differentiation and breeding range: *T. c. cyanurus* in Northern Eurasia including N Japan; *T. c. rufilatus* in C and E Himalayas and southwest China, and the proposed *T. c. pallidior* exclusively in northwest Himalayas that is sometimes lumped into *T. c. rufilatus* (Dickinson 2003; del Hoyo et al. 2005). Most recently the nominate race and *T. c. rufilatus* have been regarded as two distinct species based on their plumage, song-types, and distribution (del Hoyo et al. 2005; Rasmussen and Anderton 2005; Robson 2008). The two taxa are morphologically prominent in plumages, that is, more dark blue pigmentation of the adult males and the greyer pigmentation of the females and immatures in *T*.* c*. *rufilatu*s than in *T*.* c*. *cyanurus* (Fig. 1). More *T*.* c*. *rufilatu*s has bright blue in brows rather than white in the latter taxon (Fig. 1). Ecologically, the former taxon is a short-distance altitudinal migrant, whereas *T*.* c*.* cyanurus* is a long-distance migrant traveling between Siberia and south China. No overlap breeding zone is found between *T*.* c*. *cyanurus* and *T. c. rufilatus* (including *T. c. pallidior*). Though substantial differentiation in several aspects (including plumage, song-types, and allopatric distribution) indicates the two subspecies may merit separate species status, no genetic evidence has been presented (del Hoyo et al. 2005; Rasmussen and Anderton 2005; Robson 2008).

The allopatric distribution of the Red-flanked Bluetail resembles the pattern of North Palearctic and Sino-Himalayas within several passerine species complex, attributed to climatic and vegetation changes since the Pliocene (Päckert et al. 2009, 2012; Price 2010; Martens et al. 2011). We assume that similar biogeographic patterns may reflect a consensus evolutionary history, and thus rationally hypothesized that the general phylogeographic pattern of the Bluetail complex possibly followed the divide between Northern Palearctic and Sino-Himalayan taxa given the distributional and ecological evidence mentioned above. However, species-specific phylogeographic patterns have to been carefully investigated using rigorous genetic approaches because random nongenetic variations in morphology and migratory strategies within a species (Rolshausen et al. 2009; Liu et al. 2012) can evolve very fast than neutral genetic markers (Zink and Barrowclough 2008).

Moreover, another main goal of the present study is to investigate taxonomic status and species delimitation of the Red-flanked Bluetail. Species delimitation also strongly relies on the application of species concepts (Mayr 1942; Cracraft 1989; Andersson 1990). In fact, the intent of most species concepts shares a fundamental idea that a species in nature is an evolutionary distinct lineage (de Queiroz 2007). So to define a species, we identify separately evolving lineages based on monophyly and diagnosability under the phylogenetic species concept (Cracraft 1989) as well as the well-known biological species concept (Mayr 1942). We applied these criteria to elucidate the species limits in the Red-flanked Bluetail complex in case of genetic analysis based on two Z-linked fragments and two mitochondrial genes. The Z-linked loci are considered to be involved in species recognition traits and may be important for genes involved in reproductive isolation in birds (Sæther et al. 2007). Meanwhile, compared with nuclear genes, mtDNA can provide similar but more sensitive genetic information about the divergence of closely related taxa (Zink and Barrowclough 2008). Furthermore, considering similar phylogeographic pattern of North Palearctic region and Sino-Himalayas within several passerine birds in previous studies (Päckert et al. 2009, 2012; Price 2010; Martens et al. 2011), molecular dating between the two taxa of Red-flanked Bluetail was also approximately estimated based on a common mitochondrial clock (cytochrome b). All genetic markers and analysis methods employed in this study have been proved to be effective and enough to species delimitation and phylogenetic analysis (Backström et al. 2006; Zink and Barrowclough 2008; Päckert et al. 2009, 2012; Martens et al. 2011; Wu et al. 2011).

## Materials and Methods

### DNA sampling and sequencing

**Figure 2:**
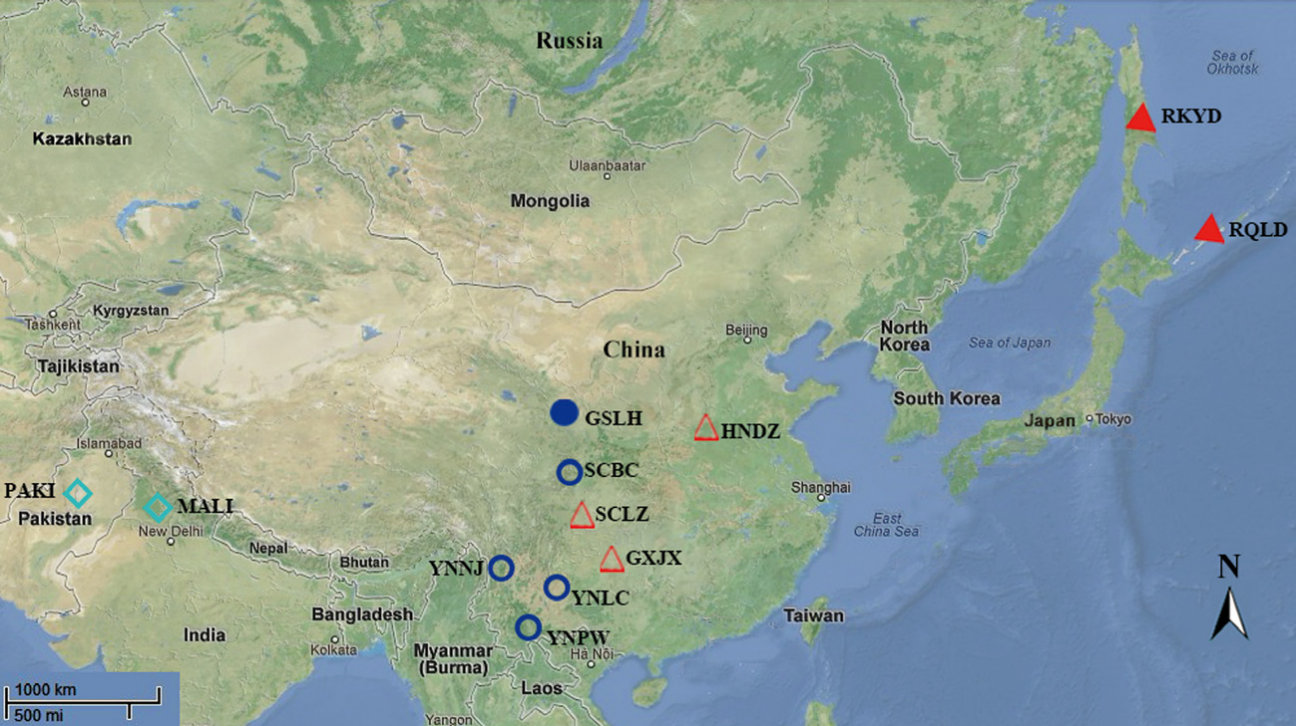
Sampling of *Tarsiger cyanurus* complex (For the abbreviation of populations see Table 1). Solid symbols are breeding regions and hollow are wintering regions for *T*.* c*.* cyanurus* (red) and *T*. *c*. *rufilatus* (dark blue). *T*.* c*.* pallidior* are light blue and samples from nonbreeding seasons.

Tissue samples were collected from our field sampling trips and museum tissue collection. Because both *T*.* c*. *cyanurus* and *T*.* c*. *rufilatus* are migrants, we included samples of these two taxa from both the breeding and nonbreeding ranges (Fig. 2 and Table 1). They were two breeding and three nonbreeding localities of *T*.* c*. *cyanurus* (32 individuals, including 17 females, nine males, and six sex unidentified individuals), one breeding and four nonbreeding localities of *T*.* c*. *rufilatus* (28 individuals, including 11 female, nine males, and eight sex unidentified individuals), and two sex unidentified individuals from two nonbreeding localities of *T*.* c*. *pallidior*. The homologs sequences of congenus species, the Golden Bush-robin (*Tarsiger chrysaeus*), and related genus species, the Plumbeous Water-redstart (*Rhyacornis fuliginosus*) were sequenced as outgroups to root the phylogenetic tree (Sangster et al. 2010).

**Table 1:** *Tarsiger cyanurus* samples used in this study.

				GenBank Accession Numbers			
Subspecies	Population	Locality	Sample sizes	ctyb	ND2	GPBP1	NNT
*T. c. cyanurus*	RQLD	S. Kurile Is., Iturup I., Russia	6	KJ024109–KJ024114	KJ024173–KJ024178	KJ024237–KJ024242	KJ024299–KJ024304
	RKYD	Sakhalin I., Dolinsk & Noglicky District, Russia	7	KJ024115–KJ024121	KJ024179–KJ024185	KJ024243–KJ024249	KJ024305–KJ024311
	HNDZ	Dongzhai, Henan, China	10	KJ024122–KJ024131	KJ024186–KJ024195	KJ024250–KJ024259	KJ024312–KJ024321
	GXJX	Jinxiu, Guangxi, China	4	KJ024132–KJ024135	KJ024196–KJ024199	KJ024260–KJ024263	KJ024322–KJ024325
	SCLZ	Luzhou, Sichuan, China	5	KJ024136–KJ024140	KJ024200–KJ024204	KJ024264–KJ024268	KJ024326–KJ024330
*T. c. rufilatus*	YNPW	Pingbian, Yunnan, China	6	KJ024141–KJ024146	KJ024205–KJ024210	KJ024269–KJ024274	KJ024331–KJ024336
	YNNJ	Nujiang, Yunnan, China	7	KJ024147–KJ024153	KJ024211–KJ024217	KJ024275–KJ024281	KJ024337–KJ024343
	YNLC	Lincang, Yunnan, China	8	KJ024154–KJ024161	KJ024218–KJ024225	KJ024282–KJ024289	KJ024344–KJ024351
	SCBC	Beichuan, Sichuan, China	3	KJ024162–KJ024164	KJ024226–KJ024228	KJ024290–KJ024292	KJ024352–KJ024354
	GSLH	Lianhua Mountain, Gansu, China	4	KJ024165–KJ024168	KJ024229–KJ024232	KJ024293–KJ024296	KJ024355–KJ024358
*T. c. pallidior*	MALI	Manalind, India	1	KJ024169	KJ024233	KJ024297	KJ024359
	PAKI	Pakistan	1	KJ024170	KJ024234	KJ024298	KJ024360

Genomic DNA was extracted from tissues using the method described by Zhang and Hewitt (1998). Two mitochondrial genes (cytb and the second subunit of nicotinamide adenine dinucleotide dehydrogenase [ND2]) and two nuclear Z-linked fragments (GC-rich promoter binding protein 1 gene intron [GPBP1] and nicotinamide nucleotide transhydrogenase intron [NNT]) were amplified and sequenced in both directions with published primers and PCR protocols (Hackett 1996; Johnson and Sorenson 1998; Backström et al. 2006; Li et al. 2006; Table 2). The resulting sequences of *T*.* c*. *cyanurus* and *T*.* c*. *rufilatus* have been deposited in GenBank (Accession No. cytb, KJ024109–KJ024170; ND2, KJ024173–KJ024234; GPBP1, KJ024237–KJ024296; NNT, KJ024299–KJ024358). But for *T*.* c*. *pallidior*, only four sequences of two mitochondrial genes were obtained from Professor Trevor D. Price, and no nuclear fragments were available. DNA sequences of *T. chrysaeus* and *R. fuliginosus* were also deposited in GenBank (Accession No. cytb, KJ024171–KJ024172; ND2, KJ024235–KJ024236, GPBP1, KJ024297–KJ024298; NNT, KJ024359–KJ024360).

**Table 2:** Primer pairs used for the amplification and sequencing of *Tarsiger cyanurus*.

Locus	Primer	Primer sequence (5′–3′)	References
cytb	L13653	TAGGATCTTTCGCCCTATC	Li et al. (2006)
	H14296	TTGTTTGATCCTGTTTCGTG	Li et al. (2006)
	L14192	CCTAGTAGAATGACTATGAGG	Li et al. (2006)
	H14853	TTACAAGACCAATGTTTTTATA	Li et al. (2006)
ND2	L5215	TATCGGGCCCATACCCCGAAAAT	Hackett (1996)
	H5766	CTCTTATTTAAGGCTTTGAAGGC	Johnson and Sorenson (1998)
	L5758	GGCTGAATRGGMCTNAAYCARAC	Johnson and Sorenson (1998)
	H6313	GGATGAGAAGGCTAGGATTTTKCG	Johnson and Sorenson (1998)
GPBP1	Forward	CTTTTGTGGACGGAGAATCG	Backström et al. (2006)
	Reverse	ATTTCTGCCTTGTGAACGCC	Backström et al. (2006)
NNT	Forward	GCTGAAATGAAACTCTTTGC	Backström et al. (2006)
	Reverse	TCCACAACAACTGAACCTTC	Backström et al. (2006)

### Genetic diversity

The number of segregating sites, haplotypes, and base substitutions of cytb, ND2 and their combined sequences (mitochondrial DNA, mtDNA) were estimated on *T*.* c*. *cyanurus*, *T*.* c*. *rufilatus,* and/or *T*.* c*. *pallidior* by DnaSP 5 (Librado and Rozas 2009) and MEGA 5 (Tamura et al. 2011). The estimation of standard genetic diversity indices, including the haplotype diversity (*h*) and nucleotide diversity (*π*) and the neutrality tests, that is, Tajima's *D* and Fu's *Fs* were carried out in DnaSP 5. We also calculated the number of polymorphic sites and the number of genotypes of the unphased sequences of Z-linked loci, GPBP1, NNT, and their combined sequences (zDNA) by MEGA 5.

### Lineage divergence analysis

The TPM2uf+I+G and TPM3uf+I+G model for the mtDNA and zDNA were selected, respectively, by the Akaike information criterion in jModelTest 0.1.1 (Posada 2008). Using these models, phylogenetic reconstruction based on the mitochondrial and Z-linked (excluding the indels) data set was carried out using maximum-likelihood (ML) trees and Bayesian inference (BI) approaches. The ML trees were constructed with PhyML 3.0 (Guindon et al. 2010) using approximate likelihood-ratio test (aLRT) branch supports (Anisimova and Gascuel 2006) to estimate the reliability of each node. For BI analysis, two independent runs of Markov chain Monte Carlo (MCMC) were launched for 1.0 × 10^7^ generations and sampled every 1000 generations by MrBayes 3.12 (Ronquist and Huelsenbeck 2003), and the rest parameters were kept as default settings, respectively. The stability of two runs and convergence of the MCMC were checked with Tracer 1.5 (Rambaut and Drummond 2007). The first 25% of samples were discarded as burnin, and the remaining saved samples were used to estimate posterior probabilities (PP) of each bipartition. These phylogenetic trees were viewed by FigTree V. 1.3.1 (http://tree.bio.ed.ac.uk/software/figtree/). Further, we depicted the haplotype relationships among populations of the Bluetail using the median-joining (MJ) networks in Network 4.5.1.0. (http://www.fluxus-engineering.com/sharenet.htm).

The average *P*-distance within *T*.* c*. *cyanurus*, *T*.* c*. *rufilatus,* and/or *T*.* c*. *pallidior* and the net distance among/between them were also computed based on mitochondrial DNA with MEGA 5. Due to lack of fossil calibrations for passerine birds, the commonly assumed average mutation rate of cytb (1.035 × 10^−8^ substitutions/site/year, s/s/y) was applied (Weir and Schluter 2008). Its conservative confidence interval was also estimated by an extremely “slow” rate (0.6 × 10^−8^ s/s/y) and an extremely “fast” rate (1.505 × 10^−8^ s/s/y) (Weir and Schluter 2008). Divergent timings of *T*.* c*. *cyanurus*, *T*.* c*. *rufilatus,* and/or *T*.* c*. *pallidior* were estimated based on the net genetic distance of cytb between them.

## Results

### Genetic diversity and lineage diagnosability

**Figure 3:**
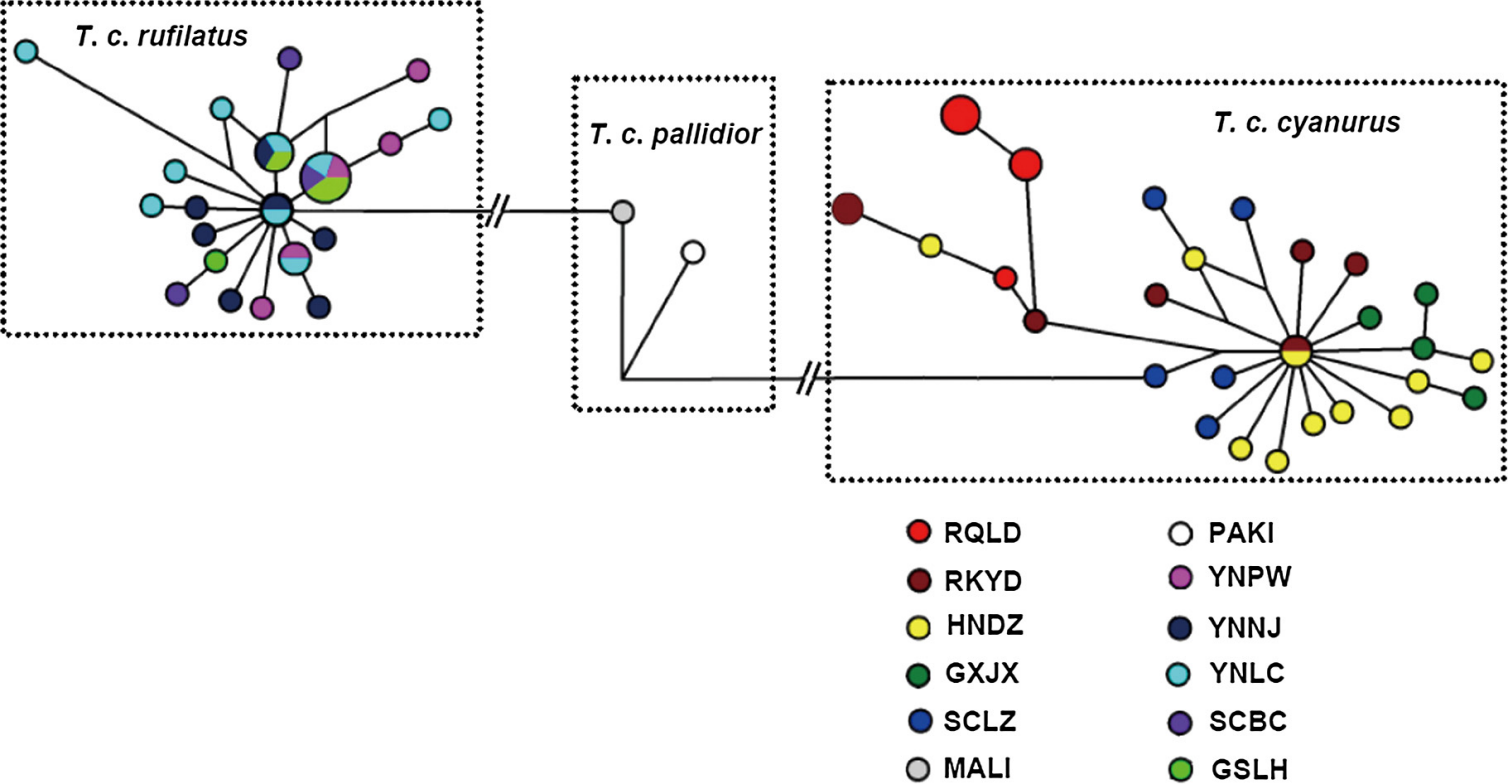
Median-joining network for 49 haplotypes based on a concatenated sequence of 1143-bp cytb and 1041-bp ND2 of 62 individuals from 12 populations of *T*.* cyanurus* complex (For the abbreviation of populations see Table 1).

A total of 62 cytb (1143 bp) and ND2 (1041 bp) sequences were obtained, and they generated 32 and 28 haplotypes, respectively. However, although cytb complete sequences were longer than ND2 complete sequences and also provided more haplotypes, ND2 yielded more variable sites (88) than that of cytb (76). With respect to *T*.* c*. *rufilatus* and/or *T*.* c*. *pallidior*, *T*.* c*. *cyanurus* has 85 diagnostic sites (fixed base sites, 33 in cytb and 52 in ND2) including five nonsynonymous sites (one in cytb and four in ND2). However, for *T*.* c*. *rufilatus* and *T*.* c*. *pallidior*, only five (including two nonsynonymous sites) and four diagnostic sites (including one nonsynonymous site) in ND2 sequences were detected, respectively. The number of haplotype, haplotype diversity (*h*), and nucleotide diversities (*π*) was higher in *T*.* c*. *cyanurus* than in *T*.* c*. *rufilatus* (Table 3). Furthermore, 62 combined sequences of two mitochondrial genes (cytb and ND2, 2184 bp), identified 49 haplotypes, and no shared haplotype was detected among/between *T*.* c*. *cyanurus*, *T*.* c*. *rufilatus,* and/or *T*.* c*. *pallidior* (Fig. 3). There were also no haplotype shared between the breeding population of *T*.* c*. *cyanurus* and *T*.* c*. *rufilatus*.

**Table 3:** Summary of nucleotide variation and demographic parameters.

Subspecies	Locus	*L*	*n*	*s*	*N*	*h*	*π*	Tajima's *D*	Fu's *Fs*
*T. c. cyanurus*	cytb	1143	32	21	18	0.925	0.003	−1.242	−9.722^***^
	ND2	1041	32	15	15	0.917	0.002	−1.171	−8.021^***^
	GPBP1	534	32	11	14	NA	NA	NA	NA
	NNT	504	32	16	12	NA	NA	NA	NA
*T. c. rufilatus*	cytb	1143	28	15	13	0.783	0.001	−2.104^**^	−9.612^***^
	ND2	1041	28	13	11	0.831	0.001	−1.807^*^	−6.047^***^
	GPBP1	528	28	33	27	NA	NA	NA	NA
	NNT	504	28	13	13	NA	NA	NA	NA
*T. c. pallidior*	cytb	1143	2	9	2	1.0	0.008	NA	NA
	ND2	1401	2	1	2	1.0	0.001	NA	NA

*n*, sample size; *L*, sequence length (bp); *s*, number of polymorphic sites; *N*, number of haplotypes for mitochondrial DNA or number of genotypes for Z-linked fragments; *h*, genetic diversity; *π*, nucleotide diversity; ^*^P <* *0.05; ^**^P <* *0.01; ^***^P <* *0.001; NA: not available.

Significantly negative values of Tajima's *D* and Fu's *Fs* tests were attributed to population expansion rather than a deviation from neutrality by selection (Table 3). No associated statistical analysis was executed for *T*. *c*. *pallidior* due to a small sample size (*N* = 2).

Also, 60 GPBP1 (534/ 528 bp) and NNT (504 bp) sequences were obtained from 32 *T*.* c*. *cyanurus* and 28 *T*.* c*. *rufilatus*. Unlike the two mtDNA genes, the two zDNA genes showed more number of genotypes in *T*.* c*. *rufilatus* (27 for GPBP1 and 13 for NNT) than in *T*.* c*. *cyanurus* (14 for GPBP1and 12 for NNT), as well as the number of polymorphic sites for GBBP1 did (33 in *T*.* c*. *rufilatus* and 11 in *T*.* c*. *cyanurus*) (Table 3). However, the number of polymorphic sites for NNT was larger in *T*.* c*. *cyanurus* (16) than in *T*.* c*. *rufilatus* (13) (Table 3). A total of 6 bp indels from three loci were found between *T*.* c*. *cyanurus* (534 bp) and *T*.* c*. *rufilatus* (528 bp) for GPBP1 in addition to two diagnostic base variation sites between them. Sixty concatenated Z-linked sequences without gaps (zDNA, 1032 bp) identified 56 haplotypes, 28 for each subspecies.

### Lineage divergence and population structure analysis

**Figure 4:**
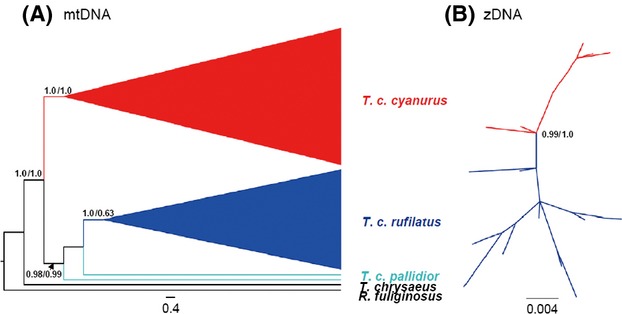
Maximum-likelihood consensus trees of *T*.* c*.* cyanurus* (red), *T*. *c*. *rufilatus* (dark blue), and *T*.* c*.* pallidior* (light blue) based on (A) mitochondrial DNA data and (B) Z-linked fragments (unrooted tree) with branch support values (approximate likelihood-ratio test/Bayesian posterior probability). Only branch support values above 0.50 are shown.

Both ML and BP trees based on the mtDNA (2184 bp) revealed two major clades with strong statistical support: *T*.* c*. *cyanurus* clade (aLRT = 1.0, PP = 1.0) and *T*.* c*. *rufilatus* and *T*.* c*. *pallidior* clade (aLRT = 0.98, PP = 0.99) (Fig. 4, A mtDNA). Furthermore, the second clade also included a substantial subclade including haplotypes from *T*.* c*. *rufilatus* (aLRT = 1.0, PP = 0.63).

The phylogenetic analysis based on zDNA (1032 bp) executed with two outgroups indicated a weakly supported paraphyletic group of *T*.* c*. *cyanurus*. Therefore, an unrooted ingroup phylogenetic tree was provided (Fig. 4, B zDNA). As with the mtDNA phylogenetic tree,both ML and BI tree of the zDNA data set also suggested two reciprocal monophyletic groups corresponding to two subspecies with significant statistic supports (aLRT = 0.99, PP = 1.0). The MJ network showed the similar topology as the phylogenetic trees (Fig. 3). The haplotypes of the mtDNA from *T. c. pallidior* was distinguished from groups of haplotypes from *T. c. cyanurus* and *T. c. rufilatus* by 98 and 11 mutation steps, respectively (Fig. 3). Further, a few frequent haplotypes were shared by individuals from different breeding or nonbreeding regions in both *T. c. cyanurus* and *T. c. rufilatus*, probably suggesting that no prominent genetic subdivision within these two taxa.

The average genetic distances for mtDNA within each clade were small: 0.003 for *T*.* c*. *cyanurus* clade and 0.002 for *T*.* c*. *rufilatus* and *T*.* c*. *pallidior* clade. However, the net genetic distance between the two clades was much more substantial: 0.039 for cytb, 0.061 for ND2, and 0.049 for mtDNA. The estimated divergent time of two clades was about 1.88 (3.25–1.30) million years ago (Mya), approximately falling into the Pliocene–Pleistocene boundary (Päckert et al. 2009).

## Discussion

Our study demonstrates the deep phylogeographic divergence between the two subspecies *cyanurus* and *rufilatus* of Red-tailed Bluetail based on both mitochondrial and Z-linked genetic markers. Such a divergence can be predated to Pliocene–Pleistocene vicariance and indicates the both subspecies merit a full species each. It is noted that our samples of *cyanurus* and *rufilatus* does not well represent their breeding range with only a limited proportion of samples from breeding grounds in Russia and Gansu and the remaining from nonbreeding range in China. This is because of logistic difficulties to visit vast and remote Siberia. However, we assume this issue could not influence our estimation of the level and tempo of divergence between *cyanurus* and *rufilatus* as field identification between the two are very obvious. But the detailed demographics and spatial substructures with these two taxa require a comprehensive coverage of the range. Nevertheless, this first phylogeographic analysis has a few implications discussed below:

### Phylogeographic pattern of *Tarsiger cyanurus* complex

The phylogeographic pattern of *T*.* cyanurus* complex is similar to some other passerine birds with Sino-Himalayan and North Palearctic split, such as *Phylloscopus fuscatus* and *P. fuligiventer,* complex and *Certhia familiaris* superspecies (Martens et al. 2011), and *Bradypterus thoracicus* and *B*. *davidi* (Alström et al. 2008). This pattern has a large divergence between taxa on/around the Tibetan Plateau and the Northern Palearctic related taxa dating back to the Pliocene–Pleistocene boundary (Johansson et al. 2007; Päckert et al. 2009, 2012; Martens et al. 2011). During this period, major vegetation-type shifts occurred on the central Loess Plateau: from dry steppe first to a temperate more humid forest ecosystem and successively to dry grassland and desert steppe until 3.7 Mya (Wang et al. 2006; Päckert et al. 2012). As small passerine birds inhabiting in forests, the deep genetic divergence between *T*.* cyanurus* and *T*.* rufilatus* might have been shaped by geographical vicariance due to grassland and desert steppe on the central Loess Plateau following the uplift of the Tibetan Plateau during the Pliocene (Wang et al. 2006; Päckert et al. 2012; Gu et al. 2013). As a result of geographical isolation and possible adaptation to different biotic environments, the two taxa began to evolve separately and diverged in several morphological and life-history traits, such as plumage, vocalizations, and migration (del Hoyo et al. 2005; Rasmussen and Anderton 2005; Robson 2008). With the augment of these divergences, the two taxa were qualified as distinct evolutionary entities.

*Tarsiger cyanurus* winters in Southeast Asia several thousand kilometers from their breeding ranges, whereas *T. rufilatus* are mostly residents or seasonally altitudinal migrants from E Himalaya to southwest China (del Hoyo et al. 2005; Rasmussen and Anderton 2005; Robson 2008). The wintering grounds of two taxa are overlapped in a small extent in Yunnan and Sichuan province, China (Zhao 2001; Y. C. Wu and F. S. Zou, unpubl. data). The breeding ranges of the two taxa are regarded as completely separated (del Hoyo et al. 2005). However, a few males of *T*.* cyanurus* form were noticed in Huzhui Mountains near Xining, the provincial capital of Qinghai on July 2008 (Y. Liu, pers. obs.), where the northern limit of *T. rufilatus* also lies in southeast Qinghai and Gansu, China (Zhao 2001). This can indicate that *T*.* cyanurus* and *T*.* rufilatus* may be slightly sympatric in this region. If so, distinct song-types and male plumages can present strong behavioral pre-mating reproductive barriers under sexual selection (Coyne and Orr 2004; Price 2010).

### Taxonomic recommendations

The present study provides the first molecular genetic analysis to delimit the taxonomic status of *T*. *cyanurus* complex. Our results suggest that three currently recognized *T*. *cyanurus* subspecies were identified to be at least two significant distinct lineages in case of mtDNA, *cyanurus,* and *rufilatus* (including *pallidior*). The two distinct genetic groups were reciprocal monophylic with substantial statistical support in the mtDNA and zDNA phylogenies. The *cyanurus* had a large number fixed base variation in mtDNA and zDNA data set with respect to *rufilatus*. Although no zDNA data of *pallidior* were available to allow genetic analysis, this may not alter the conclusions of the general pattern of major split between *cyanurus* and *rufilatus*. Further, *cyanurus* and *rufilatus* were separated from each other by large (98) mutation steps in mtDNA haplotype network. Under the phylogenetic species concept (Cracraft 1989), *cyanurus* and *rufilatus* (including *pallidior*) are reciprocal monophyly, and both have diagnosable criterion, which all show that both of them merit the level of separate species. Substantial zDNA differentiation between the two lineages is sufficient to demonstrate isolation here. These genetic characteristics are also congruent with their widely separate breeding distribution and substantial difference in male plumage, vocalizations, and migration routes (Martens and Eck 1995; del Hoyo et al. 2005; Robson 2008). Under the general lineage concept of species (de Queiroz 2007), species are delimitated based on different criteria, including morphology, ecology, behaviors, and genetic characteristics. Traits such as plumage, allopatry, vocalizations, migration routes, and reciprocal monophyly could be grouped together to define the three taxa of the Red-flanked Bluetail complex. In conclusion, this study identifies genetic distinctness of *cyanurus* and *rufilatus* based on both mtDNA and nuclear DNA. Thus, this study confirms the pervious taxonomic treatment that *T*.* cyanurus* and *T*.* rufilatus* should be two full species (del Hoyo et al. 2005; Rasmussen and Anderton 2005; Robson 2008) under both the phylogenetic and general lineage concept of species, possibly as well as biological species concept (Mayr 1942).

While *T*.* cyanurus* is monotypic species, *T*.* rufilatus* is a probably polytypic species, which includes the subspecies *rufilatus* from E Himalayas to southwest China and *pallidior* exclusively in northwest Himalayas. The *pallidior* generally resembles *rufilatus* but upper part paler upper than the later taxa, sometimes treated as a subspecies (Rasmussen and Anderton 2005) or geographical form (Dickinson 2003; del Hoyo et al. 2005). The two haplotypes of *pallidior* were grouped into the *rufilatus* clade with high statistical supports in phylogenetic-tree-based analysis. Interestingly, we found a larger average genetic distance within *pallidior* (0.008) than that within *cyanurus* (0.003) and *rufilatus* (0.001), two also group into *rufilatus* with substantial support. Several passerine birds, that is, *Certhia* [*familiaris*] superspecies and Coal Tit *Parus ater* complex, are diversified with W-E Himalayan limits along this region (Martens et al. 2011). Despite the Himalayan range plays as a hotspot for bird diversification, most of these intraspecific variations along this mountain chain were immigration rather than *in situ* speciation suggested by several phylogeographic studies (Johansson et al. 2007; Päckert et al. 2009, 2012). Genetic differentiation among Himalayan populations of most species is relatively low and apparent lack of Pleistocene divergences (Johansson et al. 2007; Päckert et al. 2009, 2012). In line with this assumption, *pallidior* may represent westward colonization of *rufilatus* given its close affiliation to the latest taxa. However, whether *pallidior* deserves a species status and its evolutionary history needs further validation through a larger data set genotyped at nuclear loci.

In conclusion, our genetic analysis corroborates the previous split of the Red-flanked Bluetail *Tarsiger cyanurus s.l*. into *T*.* cyanurus* and *T*.* rufilatus*. The divergence of the two taxa likely started around 1.88 Mya, approximately corresponding to the Pliocene–Pleistocene epoch, and such a divergence was probably associated with geographical vicariance in the central Loess Plateau. This study further suggests another independent example of the phylogeographic pattern of North Palearctic and Sino-Himalayan split in Palearctic passerine birds in which congruent patterns of divergence emerged between morphological, acoustic, distributional, and genetic evidence.
